# Cassava Intake and Vitamin A Status among Women and Preschool Children in Akwa-Ibom, Nigeria

**DOI:** 10.1371/journal.pone.0129436

**Published:** 2015-06-17

**Authors:** Fabiana F. De Moura, Mourad Moursi, Abdelrahman Lubowa, Barbara Ha, Erick Boy, Babatunde Oguntona, Rasaki A. Sanusi, Busie Maziya-Dixon

**Affiliations:** 1 HarvestPlus c/o International Food Policy Research Institute, 2033 K Street NW, Washington, DC 20006, United States of America; 2 HarvestPlus Consultant, Kampala, Uganda; 3 Federal University of Agriculture, Abeokuta, Nigeria; 4 Department of Human Nutrition, College of Medicine, University of Ibadan, Nigeria; 5 International Institute of Tropical Agriculture, PMB 5320, Oyo Road, Ibadan, Nigeria; CSIR-INSTITUTE OF GENOMICS AND INTEGRATIVE BIOLOGY, INDIA

## Abstract

**Background:**

As part of the HarvestPlus provitamin A-biofortified cassava program in Nigeria we conducted a survey to determine the cassava intake and prevalence of vitamin A deficiency among children 6-59 months and women of childbearing age in the state of Akwa Ibom.

**Methods:**

A cluster-randomized cross-sectional survey was conducted in 2011 in Akwa Ibom, Nigeria. The usual food and nutrient intakes were estimated using a multi-pass 24-hour recall with repeated recall on a subsample. Blood samples of children and women were collected to analyze for serum retinol, serum ferritin, and acute phase proteins as indicators of infection. Vitamin A deficiency was defined as serum retinol <0.70 μmol/L adjusted for infection.

**Results:**

A total of 587 households of a mother-child dyad participated in the dietary intake assessment. Cassava was very widely consumed in Akwa Ibom, mainly as *gari* or *foofoo*. Daily cassava consumption frequency was 92% and 95% among children and women, respectively. Mean (±SD) cassava intake (expressed as raw fresh weight) was 348 ± 317 grams/day among children and 940 ± 777 grams/day among women. Intakes of most micronutrients appeared to be adequate with the exception of calcium. Median vitamin A intake was very high both for children (1038 μg RAE/day) and women (2441 μg RAE/day). Red palm oil and dark green leafy vegetables were the main sources of vitamin A in the diet, with red palm oil alone contributing almost 60% of vitamin A intake in women and children. Prevalence of vitamin A deficiency ranged from moderate (16.9 %) among children to virtually non-existent (3.4 %) among women.

**Conclusion:**

Consumption of cassava and vitamin A intake was high among women and children in Akwa Ibom with a prevalence of vitamin A deficiency ranging from moderate in children to non-existent among women. The provitamin A biofortified cassava and other vitamin A interventions should focus dissemination in states where red palm oil is not widely consumed.

## Introduction

Cassava (*Manihot esculenta* Crantz), a root crop originating from South America, has become one of the major staple food crops in Nigeria since its introduction by the Portuguese before the 1900’s [1]. Nowadays, Nigeria is the highest producer of cassava worldwide with a record of 54 million metric tons produced in 2013 [2]. Akwa Ibom state, located in the South-South region of Nigeria with a population of 3,902,051, has the highest cassava consumption among the cassava producing states [3]. The cassava roots are commonly used for preparation of *gari*, edible starch, *foofoo*, *akpu*, cassava flour, and tapioca cakes [4]. *Gari* which is a granular flour made from cassava roots by peeling, washing, grating, fermenting, pressing and roasting is by far the most consumed form of cassava in Nigeria.

In terms of nutritional value, cassava is a great source of energy due to its high carbohydrate content; however, it has low levels of fat, protein and micronutrients that are essential for normal growth, eyesight and cognitive development [5]. According to the 2001–2003 Nigeria Food Consumption and Nutrition Survey the national prevalence of vitamin A deficiency (VAD) was estimated to be 29.5% among preschoolers and 13% among women of childbearing age [3]. In Akwa-Ibom state, the VAD prevalence was virtually identical to that reported at the national level for children (29.1%) but only 1% among women, while iron deficiency was 13.2% among children and 12.5% among women [3]. The high VAD prevalence among children and the high consumption of cassava prompted the selection of the state for introduction of new varieties of cassava rich in provitamin A by the HarvestPlus program as a strategy to address the need for a sustainable food based intervention for subsistence farming families in Nigeria.

HarvestPlus is part of the CGIAR Program on Agriculture for Nutrition and Health (A4NH) seeking to improve the nutrient density of staple food crops through conventional plant breeding [6]. In 2011, three first-wave varieties of yellow cassava, containing 6–7 ppm of provitamin A, were released in Nigeria, while the full target (15 ppm of provitamin A) varieties are expected to be released in 2015/2016 [7].

Initially, the target levels of provitamin A in cassava were set by using rough estimates on cassava intake (grams/day), bioconversion, the retinol equivalency of provitamin A carotenoids, and losses of provitamin A during processing and cooking [8]. However, accurate information on the above parameters were needed to confirm our initial assumptions. A study on bioconversion was performed [9] as well as retention studies. In the present study we aimed to assess the cassava intake and the vitamin A deficiency among women and preschool children in Nigeria. We conducted a cross-sectional survey in rural to moderately-urbanized areas of Akwa-Ibom, a state identified as having high cassava consumption and high VAD prevalence among children. The specific aims were: 1) to quantify the cassava and nutrient intake, and 2) to assess the vitamin A and iron status among preschool children and women of childbearing age.

## Materials and Methods

### Ethics Statement

The study was conducted in accordance with the ethical guidelines of the Declaration of Helsinki and approved by the State Health Research Ethics Committee based in the State Ministry of Health in Abuja, Nigeria. Written informed consent was obtained from the women and/or the heads of households on behalf of the women and children who participated in the study.

### Study design

The study was a cluster-randomized, cross-sectional survey conducted in the state of Akwa Ibom in Nigeria. The selection of the state was based on its vitamin A deficiency and high cassava consumption profile built using historical data from the 2001–2003 Nigeria Food Consumption and Nutrition Survey [3] as well as the prevalence of undernutrition from the 2008 Nigeria Demographic and Health Survey [10]. The target population consisted of preschool children (6–59 months) and women of childbearing age (18–49 years). The dietary, anthropometric and socio-economic components of the survey were carried out in August 2011. The households were visited a second time in October 2011 for blood collection, four months after the national vitamin A supplementation campaign.

### Sample size and sampling method

The sample size was based on an estimated 30% prevalence of low serum retinol among children as a primary outcome among children aged 6–59 months and women of childbearing age [3]. The final sample size of 660 households was calculated accepting a type I error of 0.05, a design effect of 2 and adjusting for a 15% drop out rate. The sampling procedure was a multistage cluster selection process of local government areas (LGAs) and further by enumeration areas (EAs), which is the smallest geographical cluster of households. The LGAs are grouped according to the level of urbanization (rural, moderate-urban, and urban) within each state. A comprehensive list of all EAs in selected local government areas was obtained from the State National Population Commission. Of the 31 LGAs in Akwa Ibom (16 rural, 10 moderate-urban, and 5 urban), 10 were randomly selected from the 26 non-urban LGAs, followed by the random selection of three EAs within each LGA. Therefore, each LGA comprised 3 clusters for a total of 30-cluster survey or 22 households per cluster for a total of 660 households.

### Field data collection

#### Community sensitization and subject selection

Community mobilization and sensitization was carried out in two steps: first by introducing the study to the respective LGA administration and chiefs to obtain authorization; second by holding sensitization meetings with participation of traditional leaders, representatives of local health centers and schools, heads of households and other members of the target communities. The listing and selection of households was conducted immediately after each community sensitization meeting. In each village/cluster, the research team went from house-to-house to create a list of eligible households (presence of a woman 18–49 years old and a child 6–59 months of age). Mother and child were paired for simplicity of logistics in data collection. Where a woman had more than one eligible child, the youngest child was selected as the index child for the survey.

#### Dietary Assessment

The interactive multiple-pass 24-hour recall method [11] was used to assess the usual intake of vitamin A and other nutrients. As a preparatory activity for the 24-hour recall, the inventory of foods was undertaken in the largest food market in Uyo city, the state capital, and in two towns near the ocean, Oron and Eket. The common measures used by local markets and households were also established to develop the measuring tools (i.e. direct weighing, standard size, photos, units, etc.) and the conversion factors needed for the dietary intake assessment. Further description of recipes and preparation of foods was obtained by desk review [12] and by interviewing local women. Recipes of mixed dishes were also collected in 4 out of the 30 villages that participated in the survey and the preparation of dishes according to the collected recipes occurred at least twice in each of the sampled villages. Lastly, an *ad hoc* food composition table was compiled by inputting complementary data from the Ugandan food composition table [13], the USDA nutrient database release 23 [14] the composition of West African foods [15], food composition table for the Gambia [16], and published literature of the nutrient content of individual foods consumed in Nigeria.

In the field, a two-team approach was used working in parallel to avoid the effect of interviewer fatigue and seasonal changes that could affect data quality. Each team was comprised of a team leader, one data quality editor (DQE), six enumerators, and two mother trainers. The team leader was responsible for liaising with communities, logistics management, locating the households to be included in the survey and assigning up to four households per day to each enumerator. To maximize data quality, interviewers were required to submit all questionnaires to DQE immediately after leaving the household and any existing error was corrected in the field under the DQE’s supervision. Mother trainers visited each village two days prior to the interviews to explain the 24-hour recall methodology. Plates and cups were distributed during the training to foster individual eating and therefore to increase the accuracy of individual reporting; to familiarize them with the picture charts; and to emphasize the importance of not altering their eating habits. Lastly, the 24-hour recall method was repeated a second time on a non-consecutive day for a subset of the survey sample, about 10%, in order to estimate the within-subject variation and the distribution of usual food and nutrient intake.

#### Anthropometry

Stunting (Height-for-age Z-score <-2), underweight (Weight-for-age Z-score <-2), and wasting (Weight-for height Z-score <-2) among children were estimated using WHO-Anthro software [17]. For women of childbearing age, body mass index (BMI) was calculated as weight (kg)/height (m)^2^ [18].

#### Biochemical sample collection, handling and storage

Blood samples (5 mL) were drawn from each of the children and their mothers by venipuncture at the near health facility to the survey site. Arrangements were made with the village head for the use of a community hall or the village head compound when the health facility was not available. A total of 1,270 blood samples were collected from 640 mother/child pairs. The process of sample collection was performed following standard procedures of anti-sepsis and safety. The aluminum foil wrapped vacutainer tubes with the blood samples were placed in cold boxes for about 30 to 45 min and allowed to clot after which the vacutainer tubes were centrifuged at 200 x g for 10 min to obtain clear serum. Sample processing was done in a subdued light environment. The serum samples were pipetted using Pasteur pipette into two coded Eppendorf safe-lock serum vials, wrapped in aluminum foil to protect from light. The coded, wrapped samples were placed in their respective cold boxes, which were labeled for the different laboratories. The temperature in the cold box was maintained at -4°C with frozen cooler ice pack. At the end of each day, the serum samples were transferred to the Primary Health Care clinic cold storage where they remained for a maximum period of 14 days between -35°C and -15°C. The serum samples were transported frozen to the International Institute of Tropical Agriculture, Ibadan, Nigeria where they were kept at -20°C for a period of 30 days. The samples for serum retinol, CRP and 1-AGP were air freighted in dry ice to the Medical Research Council, Cape Town, South Africa for analyses. Samples for ferritin, transferrin receptors, AGP, CRP and RBP were also air freighted in dry ice to VitMin Lab, Willstätt, Germany.

### Laboratory analysis

#### Serum retinol

Serum retinol was determined by a reversed-phase high-performance liquid chromatographic (HPLC) method with wavelength-programmed ultraviolet-visible absorbance detection [19]. The inter- and intra- batch variability was 2.5% and 3%, respectively.

#### Serum C-reactive protein and serum α1-glycoprotein

An enzyme immuno-assay for the quantitative determination of C-reactive protein (CRP) in human serum procedure was used. Commercially available external controls were used as quality measures and were obtained from BIO-RAD Clinical Diagnostics Group, BIO-RAD Liquichek Immunology Control Levels 1, 2 and 3, Ref no. 590X. The kit Cat. No. EIA-1952 was obtained from DRG Diagnostics, DRG International, USA. Alpha1-acid glycoprotein (AGP) was measured using commercially available ELISA kits no. ABIN414455, GmbH, Schloß-Rahe-Str. 15, 52072 Aachen, Germany. Commercially available external controls were used as quality measures. The inter-batch variability was 7% and intra-batch variability was 5% for both assays.

#### Retinol binding protein, ferritin, sTfR

These parameters were measured using a sandwich ELISA technique to measure vitamin A and iron status together in a small serum sample [20]. CRP and AGP as indicators of infection/inflammation were also measured using the same technique.

#### Hemoglobin

Hemoglobin was measured on site during blood collection using Hemocue-Hb 201+. The Hb 201+ system uses a modification of Vanzetti’s reagents, utilizing an azidemethemoglobin reaction yielding results within one minute. Commercially available external controls were used as quality measures.

#### Malaria

Malaria parasites density was determined by counting the number of parasitized erythrocytes per 2000 erythrocytes from a thin smear and expressed as percentage parasitaemia [21].

#### Total body iron

Body iron stores were calculated using the ratio of serum ferritin and soluble transferrin receptor values according to Cook’s equation [22].

#### Data processing and analysis

The dietary data was entered using CSDietary (SERPRO and HarvestPlus, Washington DC, 2009). A double-entry data approach was used to verify and to eliminate possible errors during this process. Statistical analysis was conducted using STATA 12 (Stata Corp., College Station, 2012). The usual food and nutrient intakes distributions were computed using the National Cancer Institute method [23]. The prevalence of inadequacy was assessed using the cut point method with estimated averages requirements established by the Institute of Medicine (IOM) [24]. For the biochemical data, the cut-off points used for each biomarker to determine adequacy are presented in Table 2. The levels of serum retinol and serum ferritin were adjusted for infection by applying the method described by Thurnham et al. [25–26], respectively.

## Results

### Participation rate and characteristics of selected households

Out of 660 households, 587 (89%) participated in the dietary assessment and socio-economic components, and 640 mother/child pairs (97%) provided blood samples. In general terms, the study population could be categorized as rural to moderately urbanized. Among the selected households, only 20% reported agriculture as their main source of income, with fishing and salaried employment as the major income generating activities in Oruk Anam and Eastern Obolo, respectively (Table 1). The conditions of the households also differed according to the LGA: 60% of the households had floors made of cement/concrete, and only in Ibeno a higher number of households (55%) had mud floors. While the majority of the households obtained their drinking water from the surface (43%) or from covered wells (40%), 10% of households reported obtaining their drinking water from piped systems.

**Table 1:** Characteristics of selected households and nutritional status of pre-school children 6–59 months and women of childbearing age from rural areas in Akwa Ibom State, Nigeria, 2011.

	Mean ± SD or %	95% CI
*n*	587	
Household characteristics		
Attained education of head of household: none or primary school only (%)	36.6	32.8, 40.6
Agriculture is primary source of income (%)	20.1	17.1, 23.6
Primary water source from pipe (%)	10.4	8.18, 13.1
Electricity at household (%)	60.5	56.5, 64.4
Cement floor in household (%)	60.3	56.3, 64.2
Access to land farming activities (%)	89.9	87.2, 92.1
Use of mosquito nets (%)	41.9	37.9, 46.0
Characteristics of children		
Age (months)	32.5 ± 14.9	30.9, 33.9
Female (%)	46.5	41.6, 51.4
*Stunting* (height-for-age Z-score)	-1.04 ± 1.8	-1.19, -0.89
Height-for-age Z-score < -2 (%)	29.7	25.9, 33.5
Height-for-age Z-score < -3 (%)	12.5	9.7, 15.2
*Wasting* (weight-for-height Z-score)	-0.08 ± 1.59	-0.21, 0.05
Weight-for-height Z-score < -2 (%)	10.4	7.9, 13.0
Weight-for-height Z-score < -3 (%)	5.5	3.5, 7.4
*Underweight* (weigh-for-age Z-score)	-0.6 ± 1.39	
Weight-for-age Z-score < -2 (%)	14.3	11.4, 17.3
Weight-for-age Z-score < -3 (%)	5.5	3.5, 7.4
Characteristics of women		
Age (years)	28.2 ± 7.9	331, 347
BMI (Body Mass Index, kg/m^2^)	24.0 ± 4.7	23.8, 24.3
Thin (< 18.4)	3.6	
Normal (18.5–24.9)	21.7	
Overweight (25–29.9)	10.4	
Obese (≥ 30)	64.3	

### Nutritional status

Overall, 29.7% of children 6–59 months of age were stunted (height-for-age Z-score <-2) (Table 1). As expected, the prevalence of stunting was highest among the youngest children, 6–11 mo (50%) and lowest among older children, 48–59 mo (22%) (data not shown). Contrary to wasting where the lowest prevalence (6.1%) was found among children 12–23 mo and the highest prevalence (14.8%) among children 48–59 mo. The mean prevalence of wasting was 10.4% (Table 1). The average BMI of women was 24.0 ± 4.7 kg/m^2^. About 21.7% were in the normal weight range, while 74.7% were categorized as overweight or obese.

### Vitamin A, iron and infection status

According to the infection stages described in Thurnham et al. 2003 [25], the majority of children (60.4%) were in some stage of sub-clinical infection as compared to 18.2% of women. Among the infected children and women, most were at early to late convalescence (chronic) (Table 2). About half of the infection could be explained by the presence of malaria parasites among children (33.9%) and women (9.68%). Infection lowers serum retinol, spuriously elevating the prevalence of vitamin A deficiency and raises serum ferritin levels, leading to an underestimation of the prevalence of iron deficiency in a population. Therefore, the unadjusted values as well as the values of serum retinol and serum ferritin adjusted for infection are presented in Table 2.

**Table 2:** Vitamin A, iron and infection status among pre-school aged children aged 6–59 months and women of childbearing age in Akwa Ibom State, Nigeria, 2011.

	Children (6–59 months)		Women of childbearing age	
	Mean or %	95% CI	Mean or %	95% CI
N	549		622	
*Infection status* ^*1*^				
CRP ≥ 10.0 mg/L (%)	27.0	23.4, 30.8	9.32	7.28, 11.9
AGP ≥ 1 g/L (%)	59.7	55.6, 63.8	14.5	11.9, 17.5
No infection (%)	39.5	35.5, 43.7	81.8	78.6, 84.7
Incubation (%)	1.82	0.992, 3.32	4.02	2.74, 5.87
Early convalescence (%)	25.1	21.7, 28.9	5.31	3.80, 7.36
Late convalescence (%)	33.5	29.7, 37.6	8.84	6.86, 11.3
Malaria parasites present (%)	33.9	30.1, 37.9	9.68	7.57, 12.3
*Vitamin A status*				
Serum retinol				
unadjusted for infection, μmol/L	0.82	0.79, 0.84	1.49	1.45, 1.60
< 0.70 μmol/L (%)	39.4	35.5, 43.4	4.06	2.79, 5.89
< 0.35 μmol/L (%)	2.75	1.70, 4.43	0.16	0.0276, 0.880
adjusted for infection, μmol/L	1.03	0.99, 1.06	1.59	1.54, 1.64
< 0.70 μmol/L (%)	16.9	13.9, 20.3	3.4	2.23, 5.14
< 0.35 μmol/L (%)	1.16	0.534, 2.51	0	0.00, 0.00
Retinol-binding protein, μmol/L	0.937	0.909, 0.965	1.72	1.67, 1.77
*Iron status*				
Hemoglobin, g/dL	9.85	9.72, 9.97	11.6	11.5, 11.8
Serum ferritin				
unadjusted for infection, μg/L	81	75.5, 86.5	59.8	55.9, 63.7
< 12–15 μg/L (%)^2^	5.1	3.55, 7.27	8.68	6.71, 11.2
< 30 μg/L (%)^3^	22.0	18.8, 25.7	-	-
adjusted for infection, μg/L	57.2	54.0, 60.4	54.6	51.2, 58.0
< 12–15 μg/L (%)^2^	6.74	4.93, 9.15	9.65	7.57, 12.2
< 30 μg/L (%)^3^	27.7	24.1, 31.6	-	-
Serum soluble transferrin receptor, mg/L	10.2	9.82, 10.6	7.92	7.61, 8.22
> 8.3 mg/L (%)	66.0	62.2, 69.6	39.1	35.3, 42.9
Iron Store, mg/kg	5.1	4.81, 5.39	5.09	4.82, 5.37

^1^CRP, C-reactive protein; AGP, α-1-acid glycoprotein; Infection status at the individual level was defined as 1) no infection: CRP < 10 and AGP < 1; 2) incubation: CRP ≥ 10 and AGP < 1; 3) early convalescence: CRP ≥ 10 and AGP ≥ 1; and 4) late convalescence: CRP < 10 and AGP ≥ 1.

^2^ < 12 μg/L indicates depleted iron stores in children less than 5 years of age, < 15 μg/L indicates depleted iron stores in individuals 5 years of age or older.

^3^ < 30 μg/L indicates depleted iron stores in the presence of infection for children less than 5 years of age.

The estimated prevalence of VAD as defined by serum retinol < 0.7 μmol/L and adjusted for infection/inflammation was 16.9% and 3.4% among children and women, respectively (Table 2). The mean serum retinol was 0.82 μmol/L for children and 1.49 μmol/L for women. Higher values were obtained for retinol-binding protein (RBP); the mean RBP was 0.94 and 1.72 μmol/L among children and women, respectively.

Seventy-five percent of children had anemia (hemoglobin < 110 g/L) while 55% of the women were anemic (hemoglobin < 120 g/L for non-pregnant women and < 110 g/L for pregnant women). The prevalence of iron deficiency as reflected by low serum ferritin adjusted for infection (< 12 μg/L for children and < 15 μg/L for women) was only 6.7% among children and 9.7% among women (Table 2). However, if we use a threshold of < 30 μg/L as recommended for children under 5 years of age in the presence of infection [26], the prevalence of iron deficiency among children increased to 22%. Furthermore, a much higher percentage of children (66%) and women (39%) had high concentration of serum transferrin receptors (> 8.3 mg/L), indicative of tissue iron deficiency.

### Dietary intake

**Fig 1:**
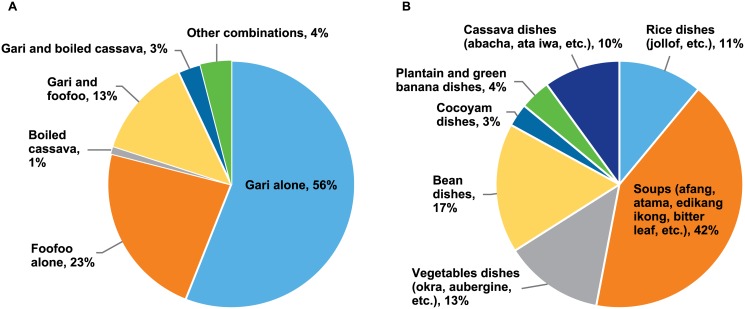
Frequency of consumption of cassava dishes (A) and frequency of consumption of dishes containing red palm oil—conditioned to red palm oil consumption (B) among pre-school children aged 6–59 months and women of childbearing age, Akwa Ibom State, Nigeria 2011.

Cassava was widely consumed in Akwa Ibom with a consumption frequency of 95%, 92% and 66% among women, children 24–59 mo, and children 6–23 mo, respectively (Table 3). The daily median intake (expressed as raw fresh weight) was also very high, 940 ± 777 grams/day for women, 348 ± 317 grams/day for children 24–59 mo, and 166 ± 234 grams/day for children 6–23 mo. Ninety-two percent of cassava was consumed in the form of *gari* and *foofoo* by women and children (Fig 1A). *Gari* alone comprised 56% of all cassava being consumed in this form with only 5% of cassava being consumed in another form, including boiled cassava. We also noticed that there was a very high consumption of red palm oil by this population with a consumption frequency of 97%, 95% and 84% among women, children 24–59 mo, and children 6–23 mo, respectively (Table 3). The red palm oil was mainly used in soups (42%), but was also used in beans- (17%), vegetables- (13%), rice- (11%) and cassava- (10%) based dishes (Fig 1B).

**Table 3:** Total usual mean intake of cassava and red palm oil expressed in grams per day among pre-school aged children 6–59 months and women of childbearing age in Akwa Ibom State, Nigeria, 2011.

Dietary Intake	Children	Children	Children	Women
	6–59 mo	6–23 mo	24–59 mo	15–49 y
	N = 584	N = 228	N = 356	N = 579
Cassava (raw fresh weight)				
All subjects	276.8 ± 301.2	165.5 ± 235.5	348.1 ± 317.0	939.8 ± 776.7
Consumers *only*	338.9 ± 300.1	249.9 ± 250.4	380.1 ± 312.3	992.9 ± 764.6 95
Consumers (%)	82	66	92	
Red Palm Oil				
All subjects	17.6 ± 14.4	13.2 ± 13.5	20.4 ± 14.3	39.1 ± 28.7
Consumers *only*	19.4 ± 14.0	15.7 ± 13.3	21.5 ± 13.9	40.4 ± 28.2
Consumers (%)	91	84	95	97

**Fig 2:**
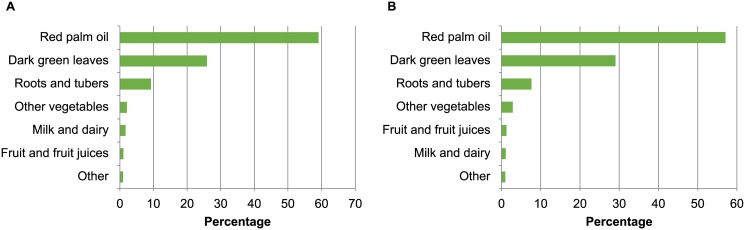
Contribution of food groups to total vitamin A intake as expressed in retinol activity equivalent among (A) women of childbearing age and (B) pre-school children aged 6–59 months, Akwa Ibom State, Nigeria 2011.

The high cassava intake was reflected in a great contribution of carbohydrate (57%) to the total energy intake for both women and children (Table 4). Overall, 30% of children and 8% of women had protein intake below the requirement. Among the micronutrients, calcium intake was inadequate among children (97%) and women (96%). The prevalence of inadequate iron intake was also very high for children (84%) and women (96%) if considered 5% bioavailability, but the inadequacy was reduced to less than half when 10% bioavailability was used. Fifty percent of women and 38% of children had inadequate intake of folate. Vitamin A intake was adequate in this population with red palm oil being the major contributor, providing almost 60% of the total vitamin A intake, followed by the intake of dark green leafy vegetables, which provided another 25 to 30% of the total vitamin A intake of children and women (Fig 2).

**Table 4:** Usual daily intakes of selected macro and micronutrients by age group.

Nutrients	Children				Women			
	EAR^1^	Median	25^th^— 75^th^ percentile	Prevalence of Inadequacy	EAR^2^	Median	25^th^— 75^th^ percentile	Prevalence of Inadequacy
*n*								
Energy (kcal)	-	1056	754–1404	-	-	2435	1976–2984	
Protein (g)	24.2^a^	34	23–49	29.9	44.6^b^	78	60–100	7.9
Fat (g)	-	36	25–50	-	-	82	65–104	
Carbohydrates (g)	-	149	108–195	-	-	346	278–428	
Vitamin A, RAE (μg)^3^	210/275	1038	682–1481	2.2	500	2441	1847–3145	< 0.1
Retinol (μg)	-	52	32–81	-	-	77	50–115	-
β-carotene (μg)	-	8070	5231–11622	-	-	19504	14491–25491	-
Vitamin B6 (mg)	0.4/0.5	0.7	0.49–0.95	17.4	1.1	1.54	1.19–1.98	18.8
Folate, DFE (μg)^4^	120/160	150	100–211	37.8	320	315	240–408	51.7
Vitamin B12 (μg)	0.7/1.0	3.2	1.9–5.1	4	2	8.1	5.6–11.4	0.8
Iron (mg)								
5% bioavailability	10.8/14.8	6.8	4.6–9.6	84.8	29.2	15.9	12.3–20.3	95.7
10% bioavailability	5.4/7.4	6.8	4.6–9.6	37.4	14.6	16.8	12.1–20.3	41
Zinc (mg)	4-Feb	4.7	3.2–6.6	11.7	7	11	8.6–14.1	11.4
Calcium (mg)	500/800	238	167–322	97.3	800	481	389–590	95.7

^1^EAR—the estimated average requirements are for children 1–3 and 4–8 years old. Estimates were calculated for each age group and an average value for all children weighted by age group, n = 506, 0.5–3 years old; and n = 78, 4–5 years old. The EAR are from IOM (2006) [25] except zinc is from IZiNCG (2004) [26] based on unrefined cereal based diet.

^2^EAR—the estimated average requirements are based on recommendations for women 19–30 years old (n = 579).

^3^ Vitamin A RAE, Retinol Activity Equivalents

^4^ Folate DFE, Dietary Folate Equivalents

^a^Based on mean weight of 27.8 kg for children (data not shown).

^b^Based on mean weight of 67.5 kg for women (data not shown).

## Discussion

This study has shown that cassava was the main staple food crop in the state of Akwa-Ibom located in the South-South region of Nigeria, which is consistent with previous findings [3], although the cassava intake of 940 ± 777 grams/day among women in our study was higher than the previously reported 700 grams/day. The higher values observed in our study may not represent a real surge in the intake of cassava over the years but could rather be due to the difference in the dietary instruments used in both surveys to capture the food and nutrient intake and in the rural focus of our survey sample. In the present study, we used the multiple-pass 24-hour recall, which was specially developed to measure the actual and usual dietary intake in rural populations of developing countries [11]. The training given to the mothers two days prior to the visit of the enumerators enabled them to better prepare for eating individually and observing their intake for an improved recall on the day of the interview. We also identified that 92% percent of the cassava consumed among women and children was in the form of *gari* and *foofoo* (56% *gari* alone, 23% *foofoo* alone, and 13% *gari* and *foofoo* combined) (Fig 1A).

In our study sample there was no vitamin A intake inadequacy (2.2% among children and < 0.1% among women). The vitamin A intake among children was estimated at 1038 μg/day, two to five times higher than those previously reported in Kenyan (378 μg/day) and Nigerian (221 μg/day) children [27]. Food sources of vitamin A were mainly food plants rich in provitamin A carotenoids, which are precursors of vitamin A. The main contributor, accounting for 50–60% of the vitamin A intake, was red palm oil, followed by 20–30% from green leafy vegetables, and less than 10% originated from dairy products, legumes, and meat (Fig 2). The palm fruit, *Elaeis guineensis*, contains on average 4,021 μg RAE/100 g of edible fruit [28]. The average consumption of red palm oil in this sample of rural children and women estimated as 20 and 40 grams/day, respectively, (Table 3), therefore the vitamin A intake from red palm oil alone, was 804 μg RAE/day among children and 1,608 μg RAE/day among women. Considering the requirements of vitamin A to be 275 μg/day for children 4–8 years of age and 500 μg/day among non-pregnant/non-lactating women, there should be no vitamin A deficiency in this population. However, 17% of the children were still considered vitamin A deficient as defined by serum retinol < 0.7 μmol/L and adjusted for infection. This discrepancy could be explained by factors that might interfere with the analysis from both sides; this includes the infection status on the biochemical side and the provitamin A degradation and bioconversion efficiency on the dietary side. It has been reported in the literature that palm fruits exposed to heat (127°C) for 35 minutes resulted in isomerization of provitamin A carotenoids and reduction in 40 to 45% of vitamin A values [28]. The red palm oil was mainly used in soups (42%), or other dishes such as beans (17%), vegetable (13%), rice (11%), and cocoyam (10%) that involved some type of heating (Fig 1B). Unfortunately, the carotenoid degradation due to cooking was not measured in this study.

As stated previously, the VAD prevalence in Akwa Ibom state was 16.9% among children 6–59 mo and 3.4% among women of childbearing age based on serum retinol values < 0.7 μmol/L adjusted for infection/inflammation. The VAD prevalence among children is considered to be of a moderate public health concern, according to the World Health Organization [29]. Had the unadjusted serum retinol values been used in our study, the VAD prevalence among children (39.4%) would have been above the 20% threshold and the vitamin A deficiency would be misclassified as of severe public health significance in this population. The presence of infection can lower serum retinol as a result of the transient decreases in the concentration of the protein that carries retinol in the blood (RBP), even in the presence of adequate vitamin A liver stores, therefore overestimating the prevalence of vitamin A deficiency. In this sample of apparently healthy children and women, 60.4% of children and 18.1% of women revealed to be at some stage of subclinical infection/inflammation, which justified the adjustment of the serum retinol values for this population [25]. Moreover, a recent WHO consultation on the use of serum retinol for assessing the VAD prevalence in population has designated vitamin A as a public health concern only when the prevalence of low serum retinol is within the specified range and another biological indicator of vitamin A status also indicates widespread deficiency or when low serum retinol that indicates widespread deficiency is combined with at least four demographic and ecological risk factors [30].

Retinol-binding protein was also measured as a proxy indicator of vitamin A status, as retinol is released from the liver in the form of a 1:1 complex of retinol and retinol-binding protein [31]. The retinol-binding protein is not photosensitive and is more stable during refrigeration, attributes that facilitate its use in field surveys. In addition, it is less expensive compared to serum retinol analysis. Although there is no consensus on the cut off value for RBP equivalent to a serum retinol concentration of < 0.7 μmol/L, there have been several studies conducted in survey subsamples in an attempt to establish the relationship between serum retinol and serum RBP for a particular population [32, 33]. For our analysis, we used the RBP cut-offs of < 0.83 μmol/L for children and < 0.78 μmol/L for women that were derived from a nationally representative sample of Cameroonian women of childbearing age and preschool children [34]. Similar to the results presented in that study, we also observed that the mean RBP was consistently higher for children (0.94 ± SD μmol/L) and women (1.72 ± SD μmol/L) than their respective mean serum retinol values of 0.82 ± SD μmol/L for children and 1.49 ± SD μmol/L for women. There was no VAD among children and women in Akwa Ibom as measured by the RBP cut-offs described above (data not shown). This finding may come as no surprise given the high vitamin A intake in this population.

The prevalence of anemia was very high among women (55%) but especially among children (75%). Unfortunately, we could not establish the type of anemia or compare our data with the most recent 2013 Nigeria Demographic and Health Survey [35], which did not include measures of anemia. Besides measuring the hemoglobin concentration, we also measured serum ferritin and serum transferrin receptor (sTfR) as the recommended approach to assess the iron status of a population [36]. According to the adjusted serum ferritin values, the prevalence of iron deficiency was fairly low, 6.7% and 9.7% among children and women, respectively, despite the very high rates of anemia. However, if we consider the values of sTfR, the prevalence of iron deficiency would be much higher, 66% among children and 39% among women. This discrepancy in the prevalence of iron deficiency has also been observed in another study in rural Burkina Faso (data not published). According to low serum ferritin, the prevalence of iron deficiency was very low, only 0–2.9% among children and 2.6–5.3% among women; however, it would be much higher, 84–91% among children and 76–79% among women, if sTfR was considered. We also note that the prevalence of anemia in rural Burkina Faso was similar to those found in our study, 72% among children and 30–46% among women.

In conclusion, the vitamin A intake in Akwa Ibom state was adequate due mainly to the high consumption of oil extracted from palm fruit that is rich in precursors of vitamin A. Still despite an adequate intake, vitamin A deficiency among children was considered of moderate public concern. The biofortified cassava program in Nigeria will disseminate yellow cassava where red palm oil is not widely consumed. Provitamin A cassava has proven to be bioavailable [9] with 30–40% retention when cooked as *gari* [37] and 82–100% when cooked as *foofoo* [38, 39]. Historically, it has been shown that not all individuals respond to a β-carotene intervention with an increase in vitamin A plasma concentrations. Those individuals are called ‘no-responders’ or ‘poor converters’. Although we have not measured the prevalence of ‘low responder’ phenotype among the study participants, evidence that consumption of yellow cassava can improve serum retinol concentrations of marginally vitamin A deficient children has recently been established in Kenya [40] where yellow cassava was proven to be efficacious and acceptable [41]. HarvestPlus is currently conducting a randomized-controlled efficacy trial in Nigeria to measure the impact of consumption of biofortified yellow cassava on serum retinol levels among children under 5 years of age.
